# Capturing variability in children’s faces: an artificial, yet realistic, face stimulus set

**DOI:** 10.3389/fpsyg.2025.1454312

**Published:** 2025-09-01

**Authors:** Sophia M. Thierry, Stefan Uddenberg, Daniel Albohn, Alexander Todorov

**Affiliations:** ^1^Department of Psychology, Brock University, St. Catharines, ON, Canada; ^2^Department of Psychology, The University of Illinois Urbana-Champaign, Champaign, IL, United States; ^3^Booth School of Business, The University of Chicago, Chicago, IL, United States

**Keywords:** children, face database, artificial face generation, expressions, emotions

## Abstract

Children’s faces are underrepresented in face databases, and existing databases that do focus on children tend to have limitations in terms of the number of faces available and the diversity of ages and ethnicities represented. To improve the availability of children’s faces for experimental research purposes, we created a novel face database that contains 500 artificial images of children that are diverse in terms of both age (ages 3 to 10) and ethnicity (representing 15 different racial or ethnic groups). Using deep neural networks, we produced a large collection of synthetic photographs that look like naturalistic, realistic faces of children. To assess the representativeness of the dataset, adult participants (*N* = 585) judged the age, gender, ethnicity, and emotion of artificial faces selected from the set of 500 images. The images present a diverse array of artificial children’s faces, offering a valuable resource for research requiring children’s faces. The images and ratings are publicly available to researchers on Open Science Framework (https://osf.io/m78r4/).

## Introduction

Face stimulus databases have become increasingly more diverse and comprehensive in recent years. These databases play a crucial role in advancing facial recognition technology and conducting research in various fields, including the study of identity and emotion perception (Bindemann et al., 2012; Marini et al., 2021; Marusak et al., 2015) and developmental and social psychology (Mather and Carstensen, 2005; Widen and Russell, 2010; Willis and Todorov, 2006). These databases provide researchers with a wide range of facial images, encompassing variations in lighting, pose, expression, and demographic characteristics, among other image and face properties. Although such databases are becoming larger and more diverse, children’s faces are often underrepresented in these databases, making it difficult or impossible to answer many kinds of research questions involving the perception and judgment of children. This work aims to improve researchers’ access to young faces by providing a diverse set of artificial child faces that vary across perceived age and ethnicity.

Traditionally, face databases have been captured in controlled conditions using standardized settings, professional models, and limited image and model variability (Beaupré et al., 2000; Belhumeur et al., 1997; Ekman and Friesen, 1976; Georghiades et al., 2001; Phillips et al., 2000). More recently, there has been a shift towards using more naturalistic and diverse datasets to better represent the real-world variability of faces. Researchers now have access to databases that provide high-resolution standardized pictures of faces of different ages, ethnicities, and emotional expressions (Bainbridge et al., 2012; Conley et al., 2018; Huang et al., 2008; Ma et al., 2015). For example, the Labeled Faces in the Wild database contains more than 13,000 images of faces collected from the internet (Huang et al., 2008). The availability of large sets of faces makes it easier for researchers interested in using face stimuli for different purposes to obtain a diverse collection of faces.

Nonetheless, even very large databases can underrepresent several demographics, making it more challenging for researchers to access nationally or globally representative face stimuli. The inclusion of children’s faces is notably limited. As images in large databases are often sourced from the internet, the images predominantly feature young adults, making it difficult to source a large subset of ethnically diverse child faces from these larger sets. Even when child faces are available (see Chandaliya and Nain, 2022), the larger face databases are usually created for training more accurate machine learning algorithms (e.g., facial recognition systems) rather than for social science research, complicating the task of curating suitable face stimuli (Huang et al., 2008; Chandaliya and Nain, 2022; Cao et al., 2018; Karkkainen and Joo, 2021; Taigman et al., 2014). Importantly, using children’s faces that are sourced from the internet poses ethical and privacy concerns, as using these images would almost certainly lack both parental consent and child assent.

The child face databases currently available for research purposes unrelated to AI training include only a limited number of faces and tend to overrepresent White faces (see Table 1 for a summary of the current landscape of child face databases). For instance, the Dartmouth Database of Children’s Faces is a controlled stimulus set including photographs of 80 White children between the ages of 6 and 16 posing eight distinct emotional expressions (Dalrymple et al., 2013). If researchers are interested in a narrower age range, this greatly reduces the number of unique faces available. The Child Affective Facial Expression Set (CAFE) is another controlled stimulus set of face photographs that includes 154 children of various ethnicities between the ages of two to eight, posing six distinct emotional expressions (LoBue and Thrasher, 2014). Despite including a more diverse sample of children’s faces, the CAFE database predominantly features White children (50%) among the relatively small number of photographs. The limited availability of diverse face sets, particularly for children, poses significant challenges to researchers seeking to study how we see, remember, or judge faces in an inclusive manner.

**Table 1:** Summary of existing child face databases.

Database	Authors	Image characteristics	Face age, gender, and ethnicity	Number of faces	Number of child faces (ages 3 to 12)
Child Affective Facial Expression Set (CAFE)	LoBue and Thrasher (2014)	Children posing seven emotional expressions on an off-white background. Children are wearing an off-white cape to cover their clothes.	90 girls and 64 boys between the ages of two and eight. The children are White (77), Black (27), Latinx (23), East Asian (16), and South Asian (11).	154	154
The Child Emotion Facial Expression Set: A Database for Emotion Recognition in Children	Negrão et al. (2021)	Children filmed and photographed posing seven emotional expressions on a blank background. Children are wearing a white shirt.	55% girls and 45% boys between the ages of four and six. The children are of White descent (71%), African descent (24%), and Asian descent (5%).	124	124
Child Emotions Picture Set (CEPS)	Romani-Sponchiado et al. (2015)	Children posing or naturally expressing six emotional expressions.	Nine girls and nine boys between the ages of six and 11. The children are White (14), Black (3) and Indigenous (1).	18	18
Children’s Spontaneous Expressions Video Database (LIRIS-CSE)	Khan et al. (2019)	Movie clips and dynamic images of children spontaneously expressing six emotional expressions with various backgrounds.	Seven girls and five boys between the ages of six and 12. The children are described as ethnically diverse.	12	12
Dartmouth Database of Children’s Faces	Dalrymple et al. (2013)	Children posing eight emotional expressions from five camera angles under two lighting conditions on a black background. Children are wearing a black bib and hat to cover clothes, hair and ears.	40 female and 40 male White models between the ages of six and 16.	80	69
National Institute of Mental Health Child Emotional Faces Picture Set (NIMH-ChEFS)	Egger et al. (2011)	Children posing five emotional expressions with direct or averted gaze on a grey background.	39 female and 20 male children between the ages of 10 and 17. Information about ethnicity was not obtained, but the authors indicate that most appear White; four girls and one boy appear non-White.	59	15
Radbound Faces Database (RaFD)	Langner et al. (2010)	Adults and children posing eight emotional expressions from five camera angles and three gaze directions on a white background. Models are wearing a black shirt.	39 White adult and 10 child models (six girls, four boys).	49	10
The University of Oregon Emotional Expression Stimulus Set (DuckEES)	Giuliani et al. (2017)	Children and adolescents posing seven emotional expressions on a white background.	22 female and 15 male models between the ages of eight and 18; 89% White.	37	10

The current database was constructed to provide the broader research community with a large database of children’s faces that are perceived to be diverse in terms of both age (ages 3 to 10) and ethnicity (representing 15 different racial or ethnic groups). Using deep neural networks, we produced a large collection of synthetic photographs that look like naturalistic, realistic faces of children varying in age and ethnicity, all without risking the privacy or dignity of any real person. The originating model was trained on thousands of faces sourced from the internet. Only images publicly released online under permissive non-commercial licenses were used in training the originating model and further authorization for the use of these images was not required (see Karras et al., 2019). Although there is a sampling bias, in that the underlying training set of internet images oversampled White adult faces, efforts were made to ensure diverse representation in the final dataset by deliberately curating images that look like children of various ethnicities. The resulting face set comprises a wide range of perceived ethnicities, including but not limited to Black, East Asian, Hispanic or Latinx, Middle Eastern, South Asian, and White. To assess the representativeness of the dataset, adult participants (*N* = 585) judged the age, gender, ethnicity, and emotion of a subset of artificial faces from the full sample of 500 images to ensure that the generated faces represented different ages and ethnicities.

## Methods

We generated 50,000 faces at random using StyleGAN2, a generative adversarial network (GAN) capable of generating highly realistic face images (Karras et al., 2019, 2020). This model was trained on over 70,000 high-quality portrait images of real faces that naturally varied along many characteristics, including age, gender, race, and expression. Face stimuli were generated using the pretrained model “stylegan2-ffhq-config-f” from NVIDIA with a truncation ψ=0.75 (for detailed information on training, hyperparameters, and various configurations, see Karras et al., 2020). Images were sampled using the W latent space (as opposed to W+ latent space).

All of the generated faces appeared realistic but occasionally contained abnormalities, such as misshapen earrings or other distorted elements within the image. The first author manually filtered the images for what looked like faces of children between the ages of 3 and 12. Images that exhibited irregularities in the facial features were discarded. The process continued until there were 80–100 artificial faces for each of five major perceived racial categories — Black, East Asian, Hispanic or Latinx, South Asian, and White. This process countered the model’s bias toward generating images of White faces. Errors in the perceived age and race/ethnicity of the images were expected, so we gathered a large sample of images and planned for further validation by other raters. Once 500 faces were collected, the first author cropped any image that had distorted features in the background. We note that some subtle abnormalities may persist in the final dataset; however, we determined these abnormalities to be unlikely to impact the overall use of the images. Only four participants from our total sample noted in the feedback section that the faces might appear AI-generated. Researchers are welcome to use the stimuli they consider subjectively appropriate, though we provide norming data for all images.

### Participants

Our final sample consisted of 585 Prolific participants from the United States (range = 18–74 years, mean = 41.82 years; www.Prolific.co). We excluded 7 participants who did not provide a completion code and eight participants who indicated that they did not take the task seriously (a rating less than 60 on a scale ranging from 1 to 100 on seriousness). Participants identified their gender as male (*n* = 333), female (*n* = 237), transwoman (*n* = 4), transman (*n* = 1), a gender not listed (*n* = 5) or preferred not to answer (*n* = 5). Participants identified their race/ethnicity as White (*n* = 394), Black (*n* = 76), two or more races (*n* = 41), Hispanic or Latinx (*n* = 31), East Asian (*n* = 14), Southeast Asian (*n* = 12), South Asian (*n* = 8), Middle Eastern (*n* = 1), Native American (*n* = 1), a race/ethnicity not listed (*n* = 4), or preferred not to answer (*n* = 3).

### Procedure

Each participant viewed a random sample of 25 unique artificial child faces from the larger set of 500 images. Five images were shown twice throughout the session to assess intra-rater reliability. Each image was rated by at least 25 participants. A post-hoc power analysis using the ICC.Sample.Size R package revealed that this sample size achieved a power of 1.00, with a significant level of *α* = 0.05 for calculating the Intraclass Correlation Coefficient (ICC; Zou, 2012). This sample size is also consistent with previous face dataset validation studies (e.g., Egger et al., 2011; Langner et al., 2010; Khan et al., 2019).

In each trial, participants viewed a face at the top of the screen and were asked to estimate the child’s age, gender, race/ethnicity, and emotional expression (angry, disgusted, fearful, happy, neutral, sad, surprised). There were 15 different response options available for participants to select from regarding race/ethnicity, as well as a response option provided for participants to input a race/ethnicity that was not among the predefined options. Participants were able to select from a dropdown menu additional information about each race/ethnicity. See S1 Appendix for the list of questions participants answered to estimate each child’s age, gender, race/ethnicity, and emotional expression.

At the end of the session, participants completed a demographic survey and answered questions about their task performance. For completing the 15-min study, participants received 1.77 GBP. The procedure was approved by the Institutional Review Board at the University of Chicago.

## Results

### Reliability

To measure intra-rater reliability, each participant rated five images twice. The Intraclass Correlation Coefficient (ICC) was used to assess agreement for the age estimate. The ICC value was 0.94, indicating excellent agreement, *95% CI* [0.93, 0.94]. Cohen’s Kappa was used to assess agreement for the categorical variables (gender, race/ethnicity, and emotion). The agreement was almost perfect for the gender estimate, ᴋ = 0.92, *Z* = 49.80, *p* < 0.001. The agreement was substantial for the emotion estimate, ᴋ = 0.72, *Z* = 57.90, *p* < 0.001. Moderate agreement was observed for the race/ethnicity estimate, ᴋ = 0.58, *Z* = 101, *p* < 0.001. With all outcomes achieving statistical significance, these results demonstrate that participants were reliable in their ratings of the four estimates.

To measure inter-rater reliability, we used ICC to assess the reliability of the age estimate. The ICC value was 0.53, indicating moderate reliability, *95% CI* [0.50, 0.56]. Given that not all faces were rated by the same number of raters, we used percent agreement to measure inter-rater reliability for the categorical variables. The percent agreement for gender was 90% (range = 0.5–1.00), 47% for race/ethnicity (range = 0.12–1.00), and 74% for emotion (range = 0.26–1.00). The percent agreement for estimates of race/ethnicity is lower due to the larger number of available response options, but still significantly above chance level (0.07), *t*(499) = 33.13, *d* = 1.48, *p* < 0.001.

### Subjective ratings

**Figure 1:**
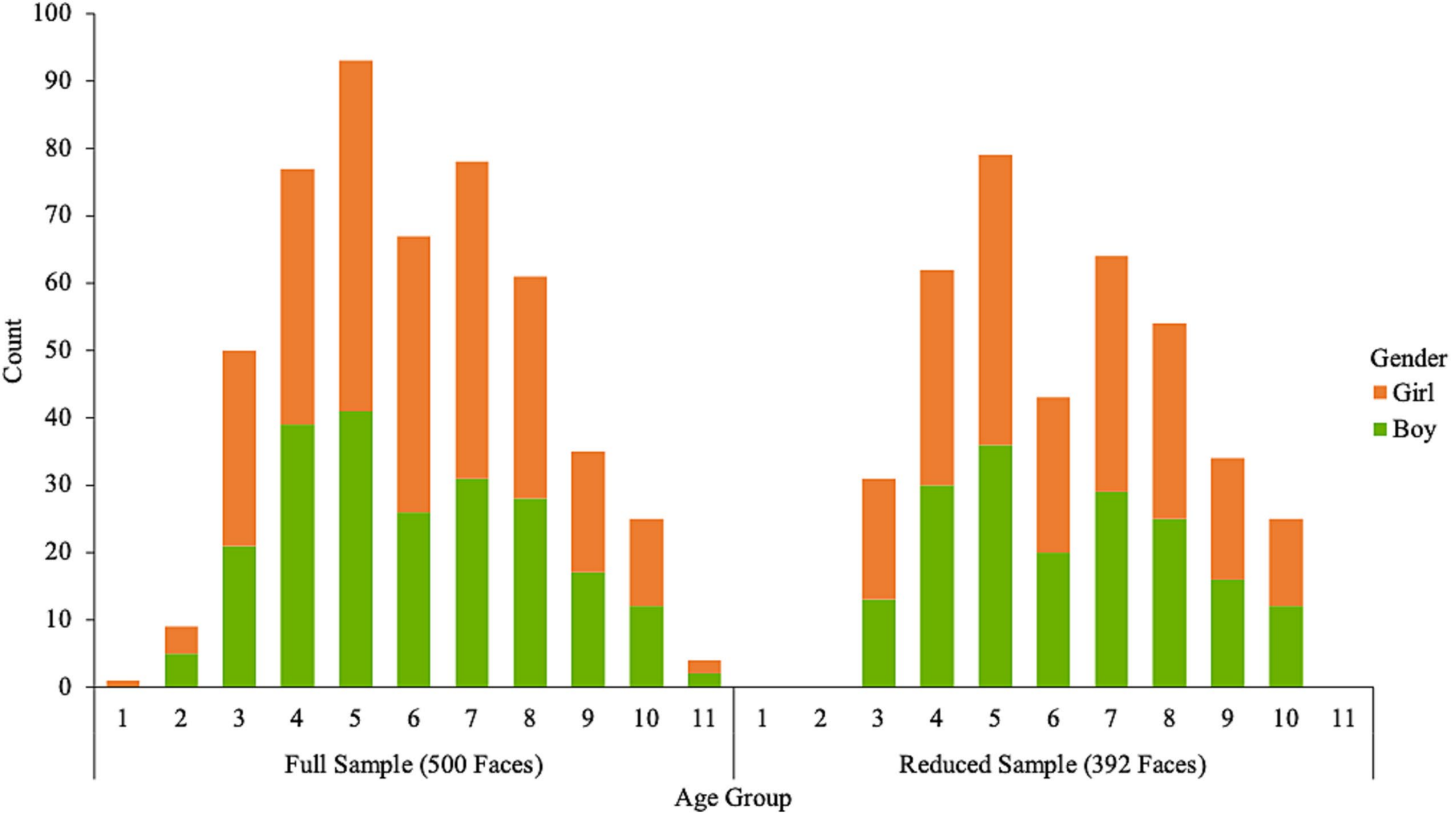
The number of perceived artificial boy and girl faces by age group and sample.

For each image, we calculated the mean age estimate and the number of participants who classified each image as a boy or girl, one or more of the 15 racial/ethnic categories, and one of the seven emotion categories. Then, we calculated the proportion of participants who classified each face as a boy or girl, one or more of the 15 racial/ethnic categories, and one of the seven emotion categories. Most faces are perceived as between the ages of 3 to 10 (*n* = 486); there are at least 12 images of boy and girl faces that fall in each of these age groups. Very few faces were perceived as over the age of 10 (*n* = 4) or under the age of three (*n* = 10). Our initial aim was to select images of children between the ages of 3 to 10, but we included the additional faces that fall outside of this age range, nonetheless. Figure 1 shows the number of perceived boy and girl faces in each age group. The top five dominant perceived races/ethnicities across the 500 faces are White (*n* = 195), Latinx (*n* = 79), Black (*n* = 77), Chinese (*n* = 39), and Filipinx (*n* = 26). The top five dominant perceived emotions are happy (*n* = 315), neutral (*n* = 151), surprised (*n* = 13), sad (*n* = 10), and happy/neutral (*n* = 5).

**Figure 2:**
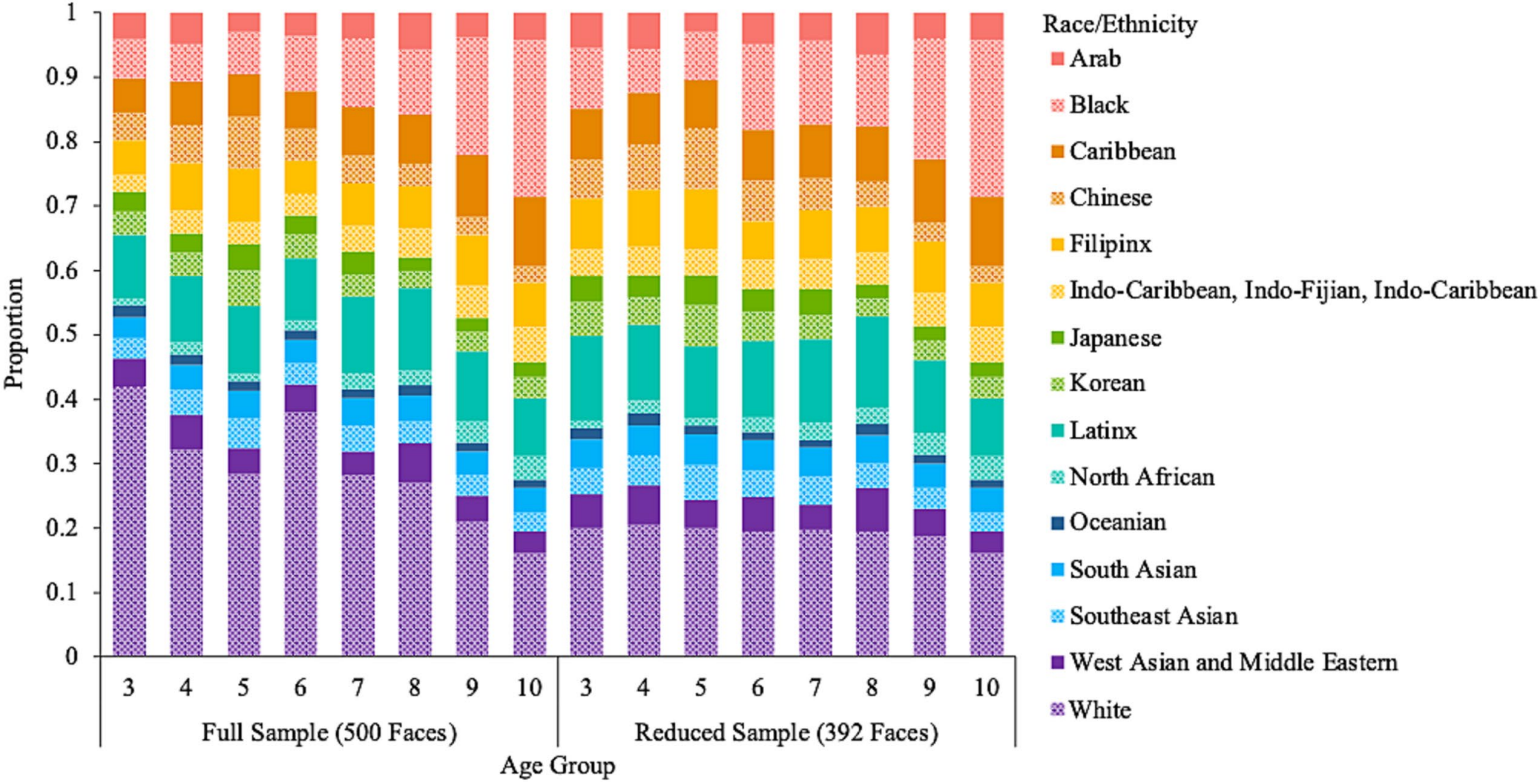
The proportion of ethnicities perceived among the artificial faces by age group and sample.

**Figure 3:**
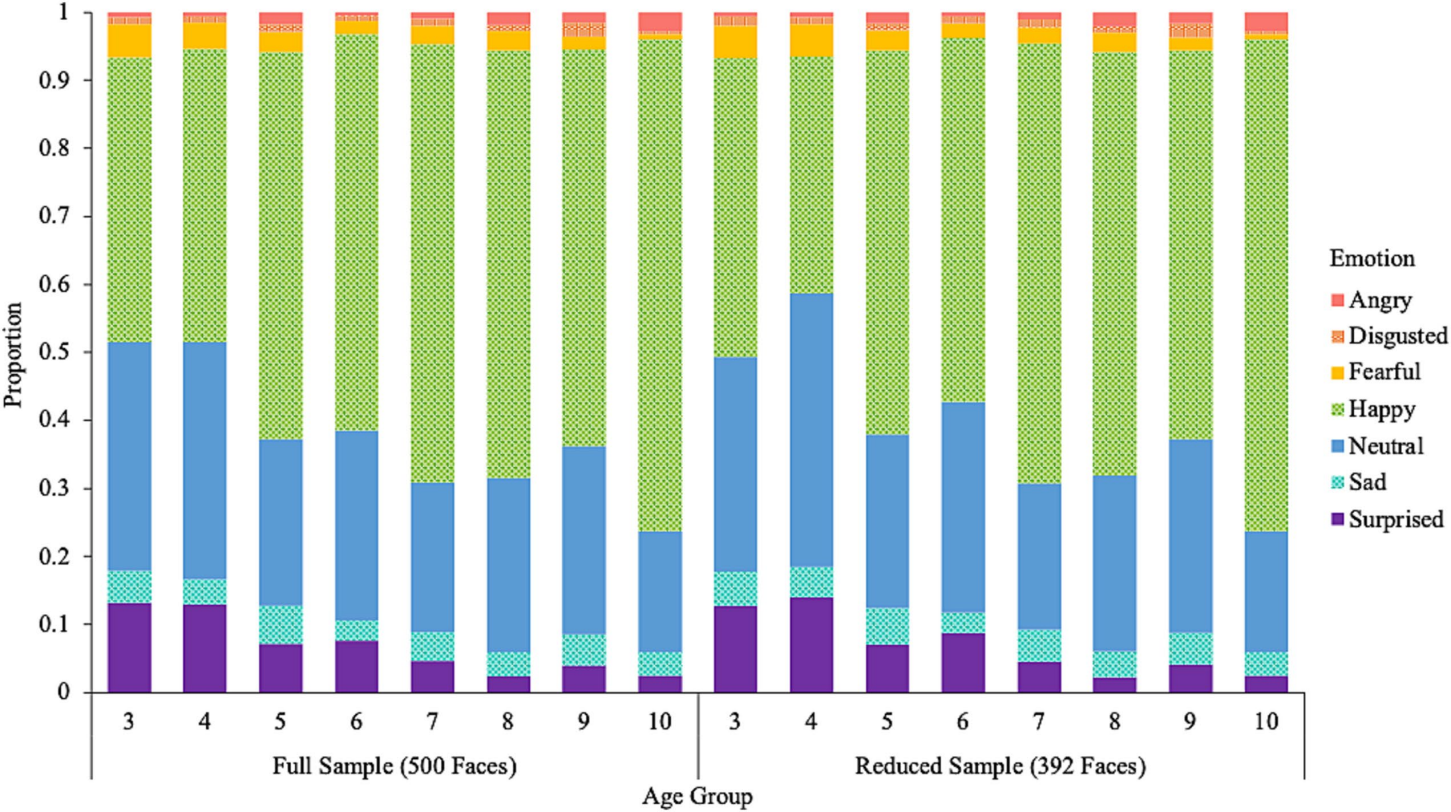
The proportion of emotional expressions perceived among the artificial faces by age group and sample.

**Figure 4:**
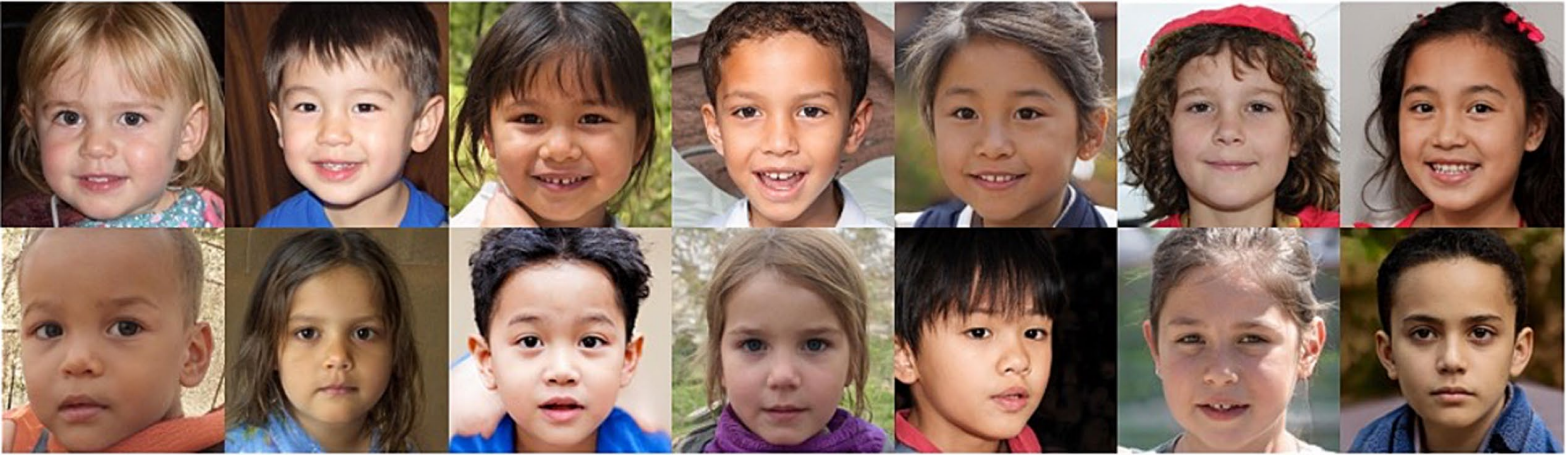
Sample images of artificial children’s faces from the artificial child face database. The faces are arranged in ascending order from perceived age three to nine (left to right), alternating girls and boys. The top row displays happy expressions; the bottom row displays neutral expressions.

To better balance the distribution of faces based on age and race/ethnicity, we created a supplemental database by excluding faces where the dominant perceived race/ethnicity was White until the proportion of faces perceived as White was no greater than 20% for each age group (3 to 10 years). This resulted in a total of 392 artificial images that are perceived to fall within the age range of 3 to 10 years. Figure 2 shows the distribution of perceived race/ethnicity by age group and Figure 3 shows the distribution of perceived emotion by age group for both the full and reduced sample of faces. Sample images of faces perceived between the ages of 3 to 10 displaying happy and neutral expressions are shown in Figure 4. Summary tables of these characteristics for the full and reduced sample can be found on our Open Science Framework page for this database.

### External validation

To further validate the ratings, we conducted an exploratory analysis to examine whether participants’ subjective ratings corresponded to attribute predictions using a generative adversarial network (GAN) (see Peterson et al., 2022). The attribute prediction model was developed from adults’ ratings of artificially generated faces from across the lifespan. We performed Spearman rank-order correlations to examine the relationship between our four attributes and the corresponding attributes in Peterson et al.’s model. For each image (*n* = 500), a prediction score was generated from Peterson et al.’s model in standard deviations. We took the mean or proportion scores from the subjective ratings and correlated these scores to the model’s prediction scores at the level of the images. We found that participants’ subjective ratings correlated to the model predictions for age (*r* = 0.32, *p* < 0.001), gender (male: *r* = 0.61, *p* < 0.001), happiness (*r* = 0.61, *p* < 0.001) and race/ethnicity (e.g., Black: *r* = 0.63, *p* < 0.001; White: *r* = 0.78, *p* < 0.001) see Supplementary Figure 1. The age correlation coefficient is likely lower than the other correlations because in our study, participants rated the age of the faces on a scale from 0 to 18, whereas the model’s predictions are based on faces spanning the entire lifespan. Since our sample only included children’s faces, the model predictions in SDs were all negative, reducing variability in the prediction score. For a complete table of the correlations between our 15 race/ethnicity classifications and the seven race/ethnicity classifications available in the model predictions, see Supplementary Table 1.

## Discussion

The Artificial Child Face Database presents 500 artificial images of (what appear to be) children of varying ages and ethnicities. After selecting a set of 500 faces generated via a deep-learning model, we conducted a validation study to ensure that the generated faces represented different ages and ethnicities.

This database provides a valuable starting point for researchers interested in using a diverse and highly variable set of child faces for their studies. While it does not present an equal representation of all age groups and ethnicities, it does offer a substantial selection of children’s faces from various perceived ethnic backgrounds, allowing researchers to select subsets of children’s faces for their research goals. In this database, many faces are perceived as more than one race/ethnicity, highlighting the importance of using a less constrained rating measure. A forced-choice task with fewer options could have led participants to make different choices (Iankilevitch et al., 2020; Nicolas et al., 2019). For this reason, we decided to visualize the findings based on the overall proportion of perceived race/ethnicity rather than the dominant race/ethnicity, as concentrating only on the dominant race/ethnicity might not provide a comprehensive representation of the faces. We recommend that researchers be transparent about how they select subsets of faces from this database and report the racial/ethnic proportions to ensure a more inclusive representation of the faces.

The current database provides an additional resource for researchers seeking face stimuli, addressing the need for greater inclusivity and representation of facial stimuli (Karkkainen and Joo, 2021; Cook and Over, 2021; Mondloch et al., 2023; Torrez et al., 2023). Existing child face databases mostly consist of tightly controlled images of White child faces (Dalrymple et al., 2013; Langner et al., 2010), and the databases that do include a more diverse range of images are limited in the number of faces available (LoBue and Thrasher, 2014; Egger et al., 2011; Giuliani et al., 2017; Khan et al., 2019; Negrão et al., 2021; Romani-Sponchiado et al., 2015). The limited availability of non-White children’s faces restricts attempts to achieve a balanced representation of ethnicity and gender in research design. For example, our research examines first impressions of faces. Only a handful of studies have examined first impressions of children’s faces. Among these studies, those that have used face stimulus sets have only used controlled images of White children (Cogsdill and Banaji, 2015; Collova et al., 2019; Eggleston et al., 2021; Ewing et al., 2019; Talamas et al., 2016; Thierry and Mondloch, 2021). With generative AI, we saw the opportunity to create a large and highly variable database for researchers to source artificial images of children’s faces. We are currently using a subset of images from this database to explore the spontaneous impressions adults and children form of children’s faces varying in emotional expression, ethnicity, and age. This is a question that we would not have been able to answer without such a large collection of images.

Another advantage of using artificial faces in research is that it reduces ethical concerns associated with using real images. For instance, although face models provide informed consent for their image to be used for research, this broad consent may not fully represent the specific ways their image will be used. Likewise, sampling faces from the internet poses additional privacy concerns, as individuals have not consented to their images being used for research purposes. These ethical concerns are particularly relevant for vulnerable populations like children who are not yet able to provide informed consent and who are not responsible for uploading their own images to the internet. These same concerns are present when using (even permissively licensed) photographs of real people sampled from the internet, whose uploaders may not realize that their images could be judged by thousands of strangers for research purposes. When using artificial faces, the resulting generated faces do not represent any specific person, ensuring individual privacy. We expect that the current dataset will be useful for research across a multitude of disciplines. For instance, in developmental psychology, these AI-generated images can be used in place of real images of children for research on peer interaction (e.g., Yazdi et al., 2020), resource allocation (e.g., Elenbaas et al., 2016), future-oriented thinking (e.g., Jerome et al., 2023), trust decisions (Grueneisen et al., 2021), and stereotyping (e.g., Shutts et al., 2016) to name a few examples.

The current database of children’s faces is only the first step in possible avenues to use artificially generated child faces in research design. Face generation software presents opportunities to create custom-made faces for specific purposes. For example, an endless array of faces of any age can be generated (Karras et al., 2019, 2020). Notably, advanced modelling techniques can be used to generate faces based on perceived physical or social characteristics, such as age or attractiveness, and to transform face photographs along these dimensions (Peterson et al., 2022; Shen et al., 2022). To date, these methods have included faces from across the lifespan, but most faces have been those of young adults. Here, we found that participants’ subjective ratings correlated with Peterson et al. (2022) model predictions for age, happiness, and race/ethnicity. Future research should explore how precisely the model’s predictions apply to child faces and the facial features or demographic characteristics that might influence the model’s accuracy. Additionally, researchers could expand on the existing models to develop models specifically for children’s faces, aiming to understand the judgments adults and children form of children’s faces. Generative models, such as Variational Autoencoders and diffusion models, are also suitable for generating child faces. The choice of which technique to use depends on the research goals (Vivekananthan, 2024). We used StyleGAN2 for image generation because it produces high-quality images and performs well with image manipulation (see Peterson et al., 2022). For instance, future work could generate child faces that appear to have a certain emotion and that vary according to specific attributes such as hair colour, “cuteness,” or “niceness,” to name just a few examples. These methods have applications for research on social impressions, emotion and identity perception, as well as social psychology more broadly.

Researchers interested in using artificially created faces should note that these faces, although realistic, might result in different conclusions than using real faces (Balas and Pacella, 2015, 2017; Miller et al., 2023; Nightingale and Farid, 2022). Although research shows that humans cannot successfully distinguish synthetic from artificially generated faces, this should be verified with our database (Nightingale and Farid, 2022; Boyd et al., 2023; Shen et al., 2021; Tucciarelli et al., 2022). Another question for future directions could be to examine whether the perceived realism of the image or image quality varies according to perceived age, gender, or ethnicity. For instance, research shows that White AI faces are perceived as human more than human faces, highlighting the importance of considering how biased training algorithms might influence how AI faces are perceived (Miller et al., 2023; Nightingale and Farid, 2022). This database can be used to broadly increase diversity in the faces sampled for research purposes; however, it should not be used to compare faces based on perceived race/ethnicity without knowing how these AI-generated faces compare to real human faces, as this may introduce biases in racial representation.

Our aim with this database is to provide a large and diverse stimulus set of child faces for researchers to use in a variety of disciplines. Researchers interested in using these faces are encouraged to share their findings and additional ratings of these images to contribute to our Open Science Platform and help expand the database further to the research community. We hope that this database makes it more accessible for researchers to use a larger and more inclusive selection of child faces in their projects.

## Data Availability

The datasets presented in this study can be found in online repositories. This data can be found here: https://osf.io/m78r4/.
