# Supplementary material for: Capturing variability in children’s faces: an artificial, yet realistic, face stimulus set
Source: Front Psychol. 2025 Sep 1;16:1454312. doi: 10.3389/fpsyg.2025.1454312 (PMC12439430; doi:10.3389/fpsyg.2025.1454312)
Supplement: Supplementary file 1 [file Data_Sheet_1.pdf]

S1 Appendix. The list of questions participants answered to estimate the child's age, gender, race/ethnicity, and emotion.

1. Gender: What is this child's gender?
    a) Boy
    b) Girl

2. Age: How old is this child?
    a) Sliding scale 0-18

3. Race/Ethnicity: What is this child's race/ethnicity? Select all that apply.
    a) Arab
    b) Black (e.g., African, African-Canadian/American, Afro-Caribbean, Afro-Latinx, etc.)
    c) Caribbean (e.g., Antiguan, Bahamian, Barbadian, Bermudan, Carib, Cuban, Dominican, Grenadian, Guadeloupean, Haitian, Jamaican, Kittitian/Nevisian, Martinican, Montserratian, Puerto Rican, St. Lucian, Trinidadian/Tobagonian, Vincentian/Grenadian, West Indian, etc.)
    d) Chinese (including Mainland China, Hong Kong, Macau, and Taiwan)
    e) Filipino/a/x
    f) Indo-Caribbean, Indo-African, Indo-Fijian
    g) Japanese
    h) Korean

i) Latino/a/x (e.g., Argentinian, Belizean, Bolivian, Brazilian, Chilean, Colombian, Costa Rican, Ecuadorian, Guatemalan, Honduran, Mexican, Nicaraguan, Panamanian, Paraguayan, Peruvian, Salvadorean, Uruguayan, Venezuelan, etc.)

j) North African (e.g., Egyptian, Libyan)

k) Oceanian (e.g., Australian, New Zealander, Pacific Islands)

l) South Asian (e.g., Bangladeshi, Pakistani, Indian, Sri Lankan, Punjabi)

m) Southeast Asian (e.g., Cambodian, Malaysian, Thai, Vietnamese)

n) West Asian & Middle East (e.g., Afghani, Armenian, Iranian, Iraqi, Israeli, Jordanian, Lebanese, Palestinian, Syrian, Yemeni)

o) White (e.g., British Isles, French, Western European, Northern European, Eastern European, Southern European, White-Canadian/American/Australian/South African)

p) I would like to specify an ethno-racial identity not listed above ____________

4. Emotion. How does this child feel?

a) Happy

b) Sad

c) Surprised

d) Angry

e) Disgusted

f) Fearful

g) Neutral
