# Supplementary material for: Capturing variability in children’s faces: an artificial, yet realistic, face stimulus set
Source: Front Psychol. 2025 Sep 1;16:1454312. doi: 10.3389/fpsyg.2025.1454312 (PMC12439430; doi:10.3389/fpsyg.2025.1454312)
Supplement: Supplementary file 2 [file Supplementary_file_1.docx]

Supplementary Material

# Supplementary Figures and Tables

**
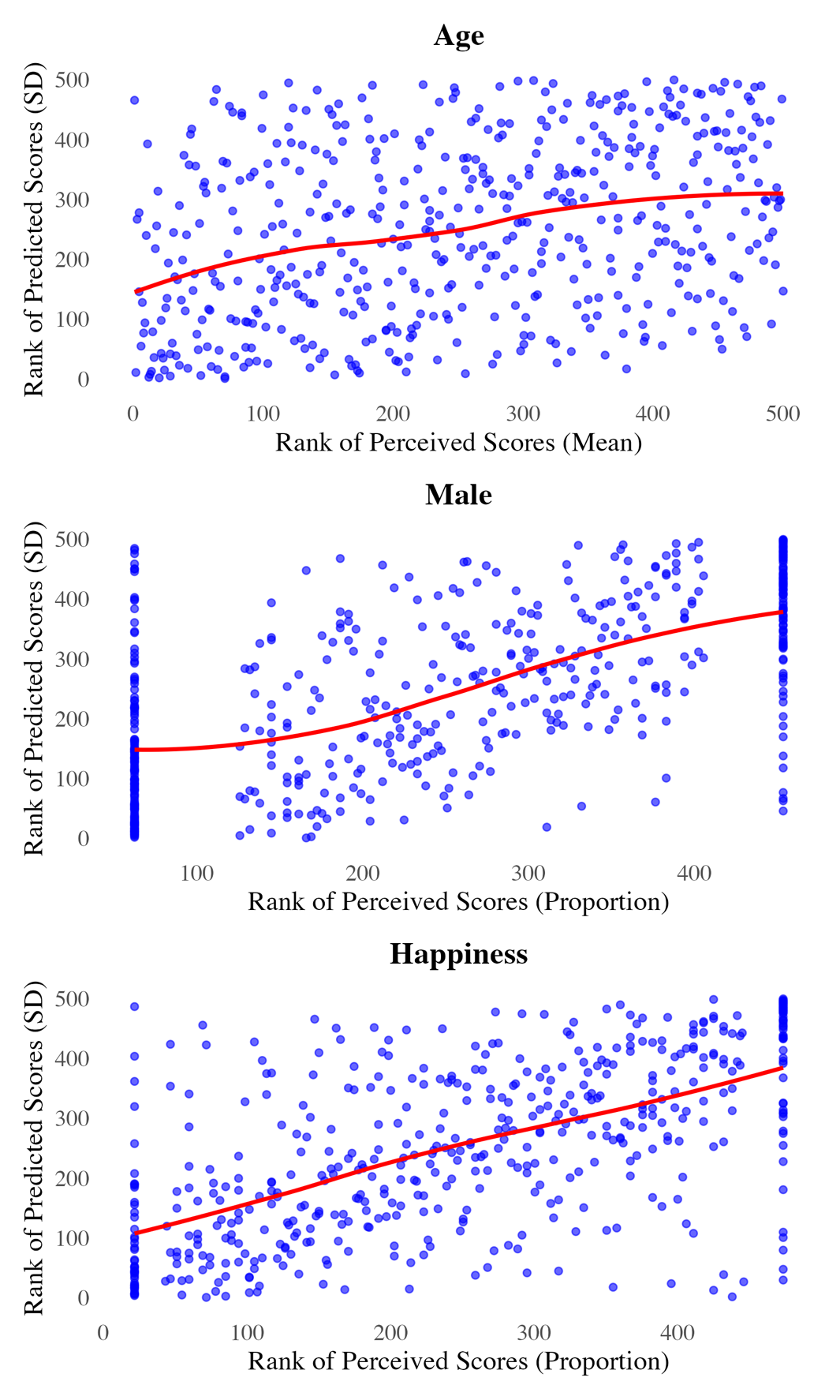
**


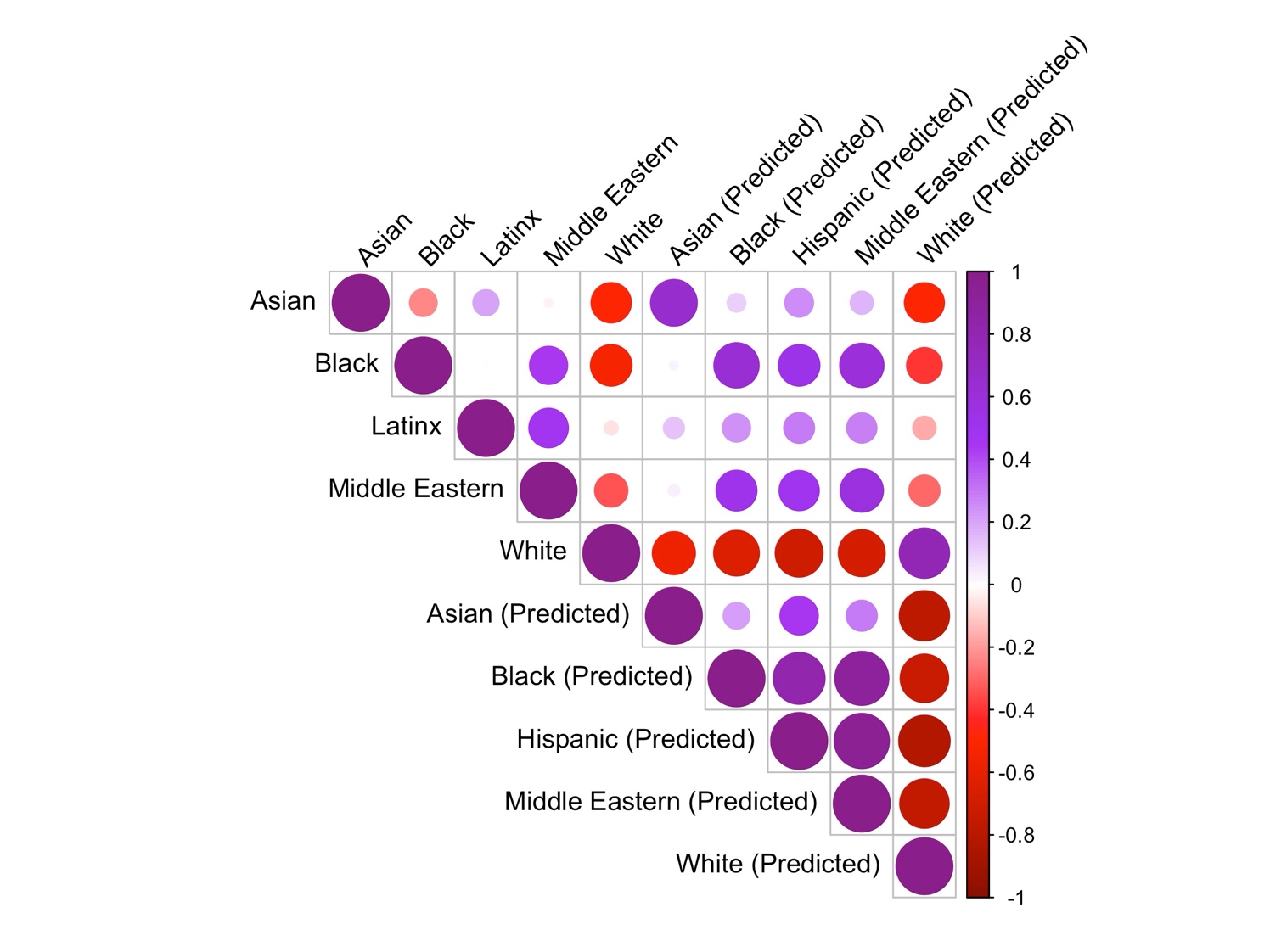
**Supplementary Figure 1.** Spearman correlation between the subjective attribute ratings and the corresponding predictions from the Peterson et al. (2022) model. Each dot represents the average of a single image’s data (*N* = 500). The ranking on the horizontal axes is based on either the mean response for that image (as in the "Age" panel) or the proportion of responses for the named category (as in the "Happy" and "Male" panels). The ranking on the vertical axes is based on the predicted response given by Peterson et al.'s (2022) model of the same attribute.

**Supplementary Figure 2.** Spearman correlation matrix showing the relationship between the proportion of participants who perceived each image (*N* = 500) as Asian, Black, Latinx, Middle Eastern, and/or White, and the corresponding race/ethnicity predictions from the Peterson et al. (2022) model: Asian (Predicted), Black (Predicted), Hispanic (Predicted), Middle Eastern (Predicted), and White (Predicted). The size and shade of the circle represent the strength of the relationship. Purple shades denote positive correlations; red shades denote negative correlations.

*Note.* To align with the race/ethnicity categories available in Peterson et al.’s prediction model, the ethnic groups were sorted into overarching categories: Asian (Chinese, Filipinx, Japanese, Korean, South Asian, Southeast Asian), Black, Latinx, Middle Eastern (Arab, North African, West Asian and Middle Eastern), White.

**Supplementary Table 1.** Spearman correlation matrix of the proportion of participants who perceived each image (*N* = 500) as one of the 15 race/ethnicity categories, and the corresponding race/ethnicity predictions from the Peterson et al. (2022) model: Asian (Predicted), Black (Predicted), Hispanic (Predicted), Middle Eastern (Predicted), and White (Predicted).

|  | 1 | 2 | 3 | 4 | 5 | 6 | 7 | 8 |
| --- | --- | --- | --- | --- | --- | --- | --- | --- |
| 1. Arab | 1 |  |  |  |  |  |  |  |
| 2. Black | 0.21*** | 1 |  |  |  |  |  |  |
| 3. Caribbean | 0.34*** | 0.68*** | 1 |  |  |  |  |  |
| 4. Chinese | -0.24*** | -0.32*** | -0.28*** | 1 |  |  |  |  |
| 5. Filipinx | 0.12** | -0.14** | 0.16*** | 0.5*** | 1 |  |  |  |
| 6. Indo-Caribbean | .12*** | 0.54*** | 0.86*** | -0.2*** | 0.26*** | 1 |  |  |
| 7. Japanese | -0.2*** | -0.36*** | -0.3*** | 0.76*** | 0.49*** | -0.21*** | 1 |  |
| 8. Korean | -0.24*** | -0.36*** | -0.26*** | 0.74*** | 0.53*** | -0.17*** | 0.79*** | 1 |
| 9. Latinx | 0.54*** | 0 | 0.27*** | -0.05 | 0.41*** | 0.25*** | -0.02 | 0.01 |
| 10. North African | 0.26*** | 0.65*** | 0.51*** | -0.32*** | -0.17*** | 0.4*** | -0.31*** | -0.31*** |
| 11. Oceanian | 0.1* | -0.05 | 0.07 | -0.13** | 0 | 0.06 | -0.12** | -0.13** |
| 12. South Asian | 0.15*** | -0.03 | 0.2*** | 0.39*** | 0.52*** | 0.28*** | 0.41*** | 0.41*** |
| 13. Southeast Asian | -0.04 | -0.21*** | 0.01 | 0.59*** | 0.63*** | 0.13** | 0.59*** | 0.62*** |
| 14. West Asian and Middle Eastern | 0.58*** | 0.17*** | 0.39*** | -0.11** | 0.21*** | 0.35*** | -0.09 | -0.09 |
| 15. White | -0.13** | -0.53*** | -0.58*** | -0.25*** | -0.42*** | -0.59*** | -0.24*** | -0.24*** |
| 16. Asian (Predicted) | -0.02 | 0.02 | 0.16*** | 0.51*** | 0.56*** | 0.25*** | 0.51*** | 0.52*** |
| 17. Black (Predicted) | 0.33*** | 0.63*** | 0.64*** | -0.12** | 0.19*** | 0.59*** | -0.14** | -0.13** |
| 18. Hispanic (Predicted) | 0.34*** | 0.53*** | 0.59*** | 0.01 | 0.31*** | 0.58*** | 0.01 | -0.02 |
| 19. Islander (Predicted) | 0.12** | 0.31*** | 0.4*** | 0.31*** | 0.49*** | 0.45*** | 0.31*** | 0.31*** |
| 20. Middle Eastern (Predicted) | 0.4*** | 0.6*** | 0.64*** | -0.08 | 0.24*** | 0.6*** | -0.09 | -0.1* |
| 21. Native (Predicted) | 0.15*** | 0.26*** | 0.37*** | 0.31*** | 0.48*** | 0.44*** | 0.32*** | 0.29*** |
| 22. White (Predicted) | -0.16*** | -0.39*** | -0.47*** | -0.26*** | -0.45*** | -0.5*** | -0.25*** | -0.26*** |
|  |  |  |  |  |  |  |  |  |
| *Note*: * *p* < .05; ** *p* < .01; *** *p* < .001 |  |  |  |  |  |  |  |  |
|  |  |  |  |  |  |  |  |  |

Table 1 Continued.

Table 1 Continued.

|  | 17 | 18 | 19 | 20 | 21 | 22 |
| --- | --- | --- | --- | --- | --- | --- |
| 1. Arab |  |  |  |  |  |  |
| 2. Black |  |  |  |  |  |  |
| 3. Caribbean |  |  |  |  |  |  |
| 4. Chinese |  |  |  |  |  |  |
| 5. Filipinx |  |  |  |  |  |  |
| 6. Indo-Caribbean |  |  |  |  |  |  |
| 7. Japanese |  |  |  |  |  |  |
| 8. Korean |  |  |  |  |  |  |
| 9. Latinx |  |  |  |  |  |  |
| 10. North African |  |  |  |  |  |  |
| 11. Oceanian |  |  |  |  |  |  |
| 12. South Asian |  |  |  |  |  |  |
| 13. Southeast Asian |  |  |  |  |  |  |
| 14. West Asian and Middle Eastern |  |  |  |  |  |  |
| 15. White |  |  |  |  |  |  |
| 16. Asian (Predicted) |  |  |  |  |  |  |
| 17. Black (Predicted) | 1 |  |  |  |  |  |
| 18. Hispanic (Predicted) | 0.83*** | 1 |  |  |  |  |
| 19. Islander (Predicted) | 0.61*** | 0.81*** | 1 |  |  |  |
| 20. Middle Eastern (Predicted) | 0.9*** | 0.94*** | 0.67*** | 1 |  |  |
| 21. Native (Predicted) | 0.54*** | 0.8*** | 0.96*** | 0.65*** | 1 |  |
| 22. White (Predicted) | -0.72*** | -0.82*** | -0.95*** | -0.75*** | -0.88*** | 1 |
|  |  |  |  |  |  |  |
| *Note*: * *p* < .05; ** *p* < .01; *** *p* < .001 |  |  |  |  |  |  |
